# Quantitative Evaluation of Composite Recyclability Using Visible-Light Microscopy and Image Processing Techniques

**DOI:** 10.3390/ma18194519

**Published:** 2025-09-28

**Authors:** Róża Dzierżak, Jolanta Sobczak, Gaweł Żyła, Jacek Fal

**Affiliations:** 1 Department of Electronics and Information Technology, Lublin University of Technology, ul. Nadbystrzycka 38 A, 20-618 Lublin, Poland; r.dzierzak@pollub.pl; 2 Doctoral School of the Rzeszow University of Technology, Rzeszow University of Technology, 35-959 Rzeszow, Poland; d569@stud.prz.edu.pl; 3 Department of Physics and Medical Engineering, Rzeszow University of Technology, al. Powstancow Warszawy 6, 35-959 Rzeszow, Poland

**Keywords:** shielding composites, digital image processing, microscope images, recycling, quantitative analysis, tungsten microparticles

## Abstract

Composites are essential materials in a wide range of industrial and medical applications due to their unique functional properties. One of the main issues of composites arises at their end-of-life stage, especially in terms of the recyclability process and its quantitative evaluation. In this study, we present a quantitative methodology for assessing the quality of composite recycling, using a paraffin-based microcomposite with the addition of tungsten particles (at one concentration 50 wt.%) as an example. Our approach combines visible-light microscopy with digital image processing techniques to obtain quantitative metrics related to recycling efficiency. The tools utilized—recognized as relatively common and uncomplicated for use in various scientific fields—have shown that the value of average particle density significantly decreased from a primary value of 43.30% to 8.30%. Consequently, the presented results confirm the usefulness of the method for the quantitative assessment of the quality of the recycling process.

## 1. Introduction

Since its discovery in the 19th century, ionizing radiation has been an incredibly fascinating phenomenon, attracting the attention of scientists, engineers, and medics. Today, it is impossible to imagine the world of science and medicine without its applications. Unfortunately, ionizing radiation is extremely harmful to living organisms [1,2,3]. Over the years, new techniques and materials have been developed to protect people, especially operators of radiation-using devices, from its effects. Historically, the first shields were made of high-mass metals, such as lead or tungsten; unfortunately, such solutions had numerous drawbacks. Lead is toxic and has a negative impact on living organisms, and these metals are also difficult to form and process. With the development of materials science, composite materials based on polymers have been developed that can be used to protect against ionizing radiation. The advancement of materials, especially nano- and microcomposites, has resulted in further breakthroughs in their application as shields, as highlighted in recent review papers [4,5,6,7,8]. However, this development has also brought new challenges, such as the disposal and recycling of composite materials.

In recent years, Sobczak et al. [9,10,11,12] have developed a new group of micro- and nanocomposite materials based on paraffin. These materials, which exhibit unique physical properties, such as easy molding using heat and the force of bare hands, could find numerous applications in industry and medicine. Furthermore, in their latest publication, Sobczak et al. [12] presented two recycling methods, based on physical and chemical approaches, where one of them allow the recovery of microparticles from composites and their reuse in a closed cycle. The recycling issue is particularly important in the context of medical applications, as these composites could be used to protect patients during therapy or diagnostic testing. Due to hygiene requirements, these materials must be sterile, which is why they are often treated as disposable products. This generates large amounts of medical waste and increases treatment costs. The possibility of recovering microparticles and nanoparticles, which constitute the main part of the cost of manufacturing such materials, would enable their reuse in new shields for subsequent patients. Therefore, a reliable and quantitative evaluation of recycling will allow for better planning of the life cycle of composites, optimization of their use, and reduction of costs, as well as negative environmental impact. As a consequence, recycling is not only a technical challenge, but also a necessity in the protection of the environment and modern medicine. To fully assess the effectiveness of the recycling process, it is necessary to quantitatively, rather than solely qualitatively, determine the number of recovered particles. In fact, even if constantly enriching the data with new composite propositions (among various areas) is vital and essential for further improvement while finding *the golden mean*, the overall issue of recycling should not be ignored. The final stage of the product’s life cycle should be the reprocessing of the waste, which not only results in saving natural resources but also paves the way to the production of novel materials.

The literature already reports the implementation of various techniques that would allow for the estimation of recycling effectiveness. Howarth et al. [13] focused on the estimation of the specific energy requirements of carbon fibre-reinforced plastic (CFRP) waste milling (bearing in mind an industrial scale) with a modelling approach. The study also included an experimental procedure using a milling machine, where the power demand was collected with a clamp meter. The outcomes showed that the mathematical model and experimental part were in good agreement (a slight difference could reflect the transient nature of the processing). All in all, the authors concluded that mechanical recycling via milling can be environmentally advantageous, as the energy required for this process (2.03–0.27 MJ/kg) is substantially lower than the embodied energy of new carbon fiber (183–286 MJ/kg). The life-cycle assessment (LCA) metrics are also considered. For example, Hervy et al. [14] analyzed the environmental impact (LCA study with various categories for instance global warming potential (GWP) in ${\mathrm{CO}}_{2}$ eq, or acidification potential (AP), in ${\mathrm{SO}}_{2}$ eq) of epoxy composites reinforced with bacterial cellulose (BC) and nanofibrillated cellulose (NFC), while neat polylactide and 30 wt.% randomly oriented glass fibre-reinforced polypropylene (GF/PP) composites constituted reference materials for a comparison. The study demonstrated that epoxy composites reinforced with BC and NFC exhibited a higher GWP indicator compared to the referenced materials, and the authors pointed to further necessary steps to create “truly green” nanocellulose-reinforced polymer composites including, e.g., a decrease in the energy required for NFC production. Nonetheless, the shielding area is not omitted; Pourzahedi et al. [15] emphasized the significance of carbon nanotube (CNT) composites in the area of electromagnetic interference (EMI) shielding applications (particularly in satellites). The study included a comparison of the life cycle energy benefits between CNT composites and the conventional solutions, where, based on the results, it was pointed out that when using CNT composites, a decrease in the mass of EMI shielding, fuel consumption, and primary energy demand was noted. It should be noted that the life cycle assessment issue is present in other sectors; for instance, in building sector [16], medicine [17], or in the production of vehicle parts [18].

Remaining on this topic, one should indicate the role of computer methods in these analyses. Indeed, it could be observed that these methods are progressively becoming an integral part of recycling analyses, such as application of convolutional neural network (CNN) tools in intelligent waste identification and recycling (IWIR) [19]. The superiority of the computed methods can be attributed to their accuracy (an ability to detect required compounds, specific features, minor impurities, and other inconsistencies in the acquired images), repeatability, and ability to process big data, along with their quick creation of comprehensive recycling reports.

Machine learning methods, including deep neural networks, have been applied for the automatic segmentation and analysis of fly ash microspheres based on images acquired with an optical microscope [20]. This approach enables the precise determination of particle size distribution and volume fraction, facilitating rapid and efficient quality control of construction materials [20]. In Zhupanska et al.’s study [21], an unsupervised machine learning method utilizing statistical distance metrics was developed for the automatic segmentation of micro-CT images of damaged CFRP composites. Similarly, in Furat et al.’s article [22], convolutional neural networks were employed to enhance grain boundaries and perform automatic segmentation of the microstructure in 3D CT data of an AlCu specimen. The method allowed accurate and efficient segmentation across the entire series of scans, even when ground truth data was only available for a single time step. In all of these examples, large datasets of image data were required to develop the methods, which are not always accessible.

This work aims to develop a tool based on microscopic image analysis to evaluate the effectiveness of microparticle recovery from paraffin-based composites in a simple, quick, and cost-effective manner. For this purpose, a computational image analysis technique is demonstrated that enables the characterization of particles and the assessment of their content within the investigated area. This is particularly useful in experimental laboratory studies where only a limited number of microscopic sample images are available. In the current study, 10 images of the sample were available before and after recycling. The image analysis enabled a quantitative assessment of the new recycling method, clearly showing its effectiveness and providing a basis for estimating microparticle recovery from paraffin-based composites.

## 2. Materials and Methods

The following subsections include information regarding sample preparation, accompanied by details of the equipment and techniques used which enabled the evaluation of the recycling process.

### 2.1. Sample Preparation

Samples, including both the shielding composite and the recycled one, were prepared in accordance with the procedures presented in Ref. [12]. Briefly, regarding the paraffin/tungsten composite, the first step included weighing the right amount of both compounds (paraffin as a base and tungsten microparticles as the shielding component—both commercially purchased). Subsequently, the sample was placed in a vacuum dryer (in order to remove air bubbles), and after crystallization, the sample was mixed using hand press for approximately 50 min. The sample was prepared in one mass fraction of tungsten particles, i.e., 50 wt.%. Regarding the preparation of the recycled sample, 1 g of composite (paraffin + 50 wt.% W) was initially placed in a vial and then immersed in a water bath (MLL 547, AJL Electronic, Cracow, Poland) set at 95 °C in order to melt the paraffin. The second step involved adding 1 g of turpentine, which was followed by mixing the vial with a shaker (IKA Vortex 3 shaker, IKA, Staufen, Germany) for 5 min. Finally, centrifugation was performed with a Jouan BR4i Multifunction Centrifuge (Thermo Electron Corporation, Waltham, MA, USA) for three minutes at a constant speed of 3000 revolutions per minute at room temperature, which resulted in the accumulation of tungsten particles at the bottom of the vial. For further analysis, the samples were placed on a standard glass slide; for the composite, a thin slice was cut from the previously prepared sample, while waste (paraffin, turpentine, and remaining particles from above the post-centrifugation-accumulated particles) was placed on a glass with a spatula. To even out the irregularities and create a uniform layer, cover glasses were added accordingly.

### 2.2. Image Acquisition

**Figure 1:**
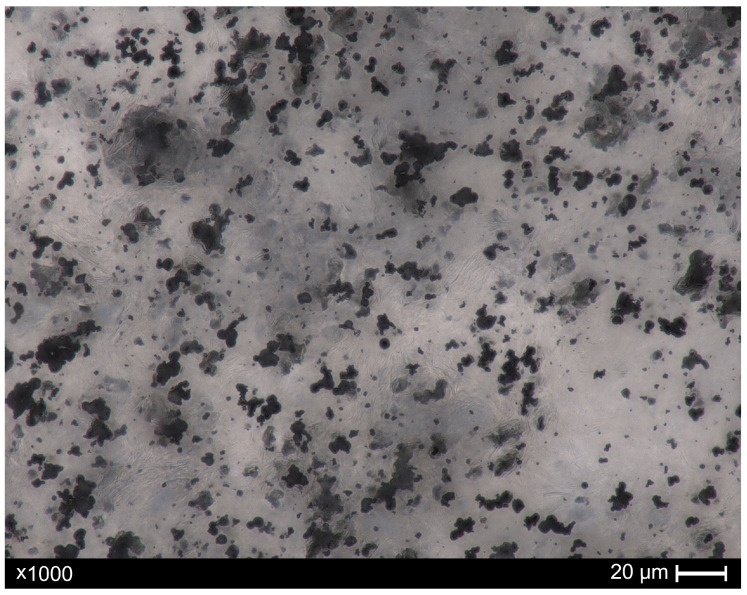
Image of a manufactured composite sample (paraffin + 50 wt.% tungsten microparticles). The black areas represent tungsten microparticles, which are dispersed among the matrix (paraffin). The image was obtained with ×1000 magnification in the transmitted light.

**Figure 2:**
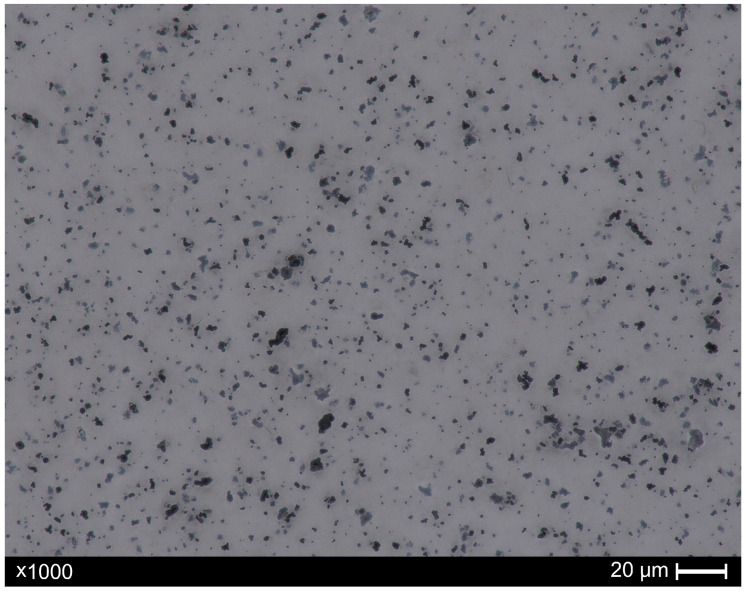
Image of sample after recycling process. The dark areas represent tungsten particles, which remained in the paraffin/turpentine mixture waste (the light area). The image was obtained with ×1000 magnification in the transmitted light.

Microscopic images of the composites, both before and after recycling, were acquired using a digital optical microscope, Keyence VHX-X1 series (Keyence, Osaka, Japan). Ten images of each sample (before and after recycling) were taken in various regions of the prepared samples. For this purpose, a microscope slide with a composite was placed on a transparent object table, allowing images to be captured in the transmitted light. All images were captured at magnifications of ×1000, with automated focus stacking capabilities. The collected pictures have a width and height of 2880 and 2160 pixels, respectively. Note that 10 pixels is equivalent to 1 µm. Representative images of the samples before and after recycling are shown in Figure 1 and Figure 2, respectively. For illustrative purposes, scale bars were added to the images. However, for subsequent image analysis (based on pixel-level processing), the original photos used for research were acquired without graphical annotations.

### 2.3. Image Processing Methods

The appropriate selection of methods for processing and analyzing microscopic images is essential to obtain reliable and reproducible results, particularly in the context of eliminating disturbances such as noise and uneven illumination. In the presented approach, a set of well-established Python (version 3.11) tools and libraries was employed to enable comprehensive image data processing. The OpenCV (version 4.11.0) library was utilized for fundamental image processing operations. NumPy provided efficient array manipulations on pixel data, while pandas was used for organizing and analyzing quantitative data extracted from the images. The scikit-image package was applied for the extraction and labeling of connected components, allowing for the identification and morphological characterization of individual structures. Additionally, visualization of the results was performed using the Matplotlib (version 3.6.3) library, facilitating the graphical representation of the analyzed particle features and the area distribution that the particles occupy in the image.

#### 2.3.1. Image Preprocessing

The initial preprocessing of microscopic images involved several critical stages designed to prepare the data for effective quantitative particle analysis. The first step consisted of converting the images to grayscale, thereby reducing the number of channels from three (RGB) to one and simplifying subsequent computational procedures. This transformation minimizes data complexity while preserving essential intensity information. In the following step, Gaussian blurring was applied by convolving the grayscale image with a 3 × 3 kernel whose weights correspond to the Gaussian function. This operation serves to smooth the image, suppress random noise, and eliminate minor acquisition-related artifacts commonly encountered in microscopic imaging. Importantly, Gaussian filtering enhances image quality without compromising the integrity of prominent object boundaries, facilitating more accurate and robust segmentation in later processing stages [23].

**Figure 3:**
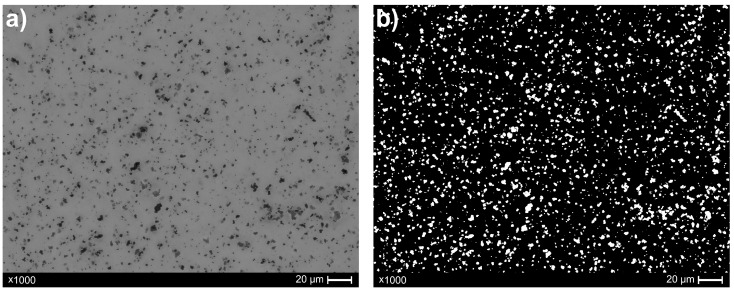
Result of applying adaptive thresholding to the image with a post-recycling sample: (**a**) original image; (**b**) image after adaptive thresholding.

Adaptive thresholding was subsequently applied following the image-smoothing step. This is a binary segmentation technique that differs from conventional global thresholding in that the threshold value is computed locally for each region of the image, rather than being based on a single global intensity value [24]. As a result, this method demonstrates robustness to non-uniform illumination and background intensity variations, which are common artifacts in microscopic or scanned images. In this study, adaptive thresholding was implemented using a mean-based approach within a 19 × 19 pixel window, from which a constant value of 5 was subtracted. This configuration enabled the precise separation of bright objects from a dark background. Additionally, an inverse-binary thresholding mode was employed, resulting in target structures (i.e., particles) being represented as bright regions against a dark background, thereby facilitating subsequent analysis and object detection. The result of this operation is shown in Figure 3.

**Figure 4:**
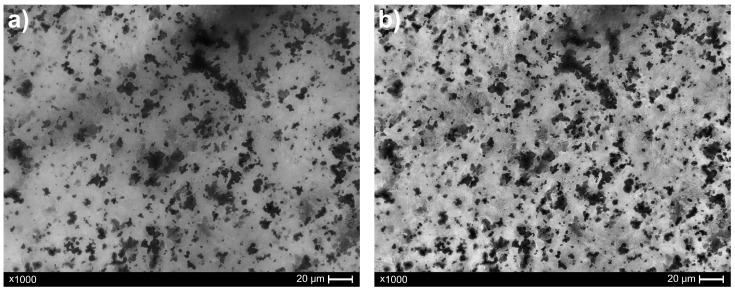
Effect of the CLAHE method on the pre-recycling composite sample image: (**a**) original image; (**b**) image after contrast enhancement.

Due to the specific characteristics of pre-recycling images, Contrast Limited Adaptive Histogram Equalization (CLAHE) was applied to enhance local image contrast (Figure 4). This technique performs adaptive histogram equalization within small, non-overlapping regions of the image, referred to as tiles. By redistributing the intensity values locally, CLAHE effectively enhances fine details, even in areas affected by uneven illumination or low contrast, without introducing the overexposure or excessive darkening often associated with traditional global histogram equalization. As a result, the processed image exhibits improved uniformity in brightness and contrast, which significantly enhances the quality of subsequent image analysis, particularly in tasks involving segmentation and object detection.

#### 2.3.2. Identification of Particles

Following the preprocessing stage, connected component labeling was applied to the microscopic images. The purpose of this method is to identify and distinguish related objects within the image. Connected component labeling involves assigning unique labels to groups of pixels that form contiguous regions, referred to as components. These regions are defined by the connectivity of pixels, with values indicating the presence of an object relative to their neighbors. This allows the algorithm to differentiate all separate connected elements in the image [25]. The resulting labels enable individual analysis of each object. Previously performed transmission electron microscopy (TEM) analysis of particles revealed an average size of 2.1 µm, with the most common occurrence being 2.5 µm; however, the analyses revealed some particles deviating from these values falling within the range of 0.5 up to 5 µm [12]. Therefore, particles with an area smaller than 25 px (which corresponds approximately to particles with a diameter not less than 0.6 µm) were excluded, which allowed for the elimination of potential minor artifacts and noise.

**Figure 5:**
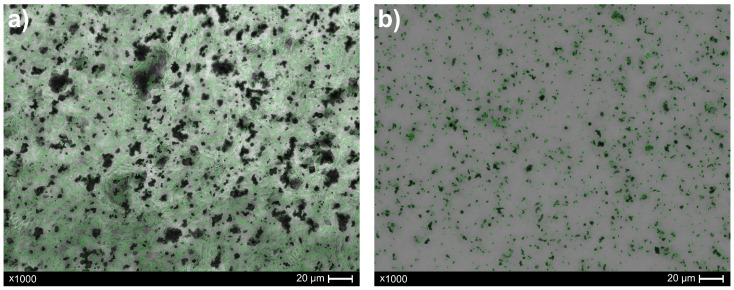
Image of sample before recycling (**a**) and after recycling (**b**) with identified particles marked.

Particle contours were delineated to support the qualitative assessment of particle morphology. A binary mask containing only the pixels of valid particles was created. The resulting contours were overlaid on the original image, converted to color space, and highlighted in green, thereby enhancing visual clarity. Below are microscopic images before and after recycling, with detected particles highlighted (Figure 5).

To provide a quantitative summary, the total image area and the cumulative area of all detected particles were computed, alongside a density metric defined as the ratio of the aggregate particle area to the total field of view area. Furthermore, particle area distribution was analyzed through the generation of area histograms, enabling the visualization of particle frequency across discrete size intervals.

## 3. Results

The following subsections present the results of microscopic image analysis of the investigated composites before and after recycling. This analysis includes the number and density of particles, as well as their area.

### 3.1. Quantitative Analysis of Particle Count and Density

For each analyzed image, both before and after the recycling process, the number of particles as well as their total area were calculated. In addition, the percentage share of the particle area relative to the total image area was determined, enabling an assessment of the effectiveness of the particle recycling method. The obtained results that are based on images acquired from different regions of the composite sample (not subjected to recycling protocol) are summarized in Table 1.

The analysis of the results obtained for ten images of the sample prior to recycling demonstrated that the number of particles remained relatively stable, ranging from 2417 to 3056 (mean: 2677). The slight variations in particle count indicate a uniform distribution of particles within the sample. In terms of the total particle area, greater variability was observed. The values ranged from 2.54 million to 2.87 million px (25,425.96–28,688.35 µ$m^{2}$), with a mean value of 2.69 million px (26,936.74 µ$m^{2}$). These findings suggest that, despite a comparable number of particles, their sizes and shapes may locally differ depending on the analyzed region of the sample. Such variability is a natural consequence of the structural heterogeneity of the material in some places, resulting from local particle agglomeration. This stems from the composites’ functionality; the samples are characterized by susceptibility to shape-change under the influence of heat and hand pressure. Consequently, modifications in the shape of the composites induce alterations in the distribution of particles within the matrix.

The particle density, defined as the percentage share of the particle area in the total image area, ranged from 40.87% to 46.12%, with a mean value of 43.30%. This mean indicates that the particles occupied slightly less than half of the analyzed image area. The highest density was recorded in region No. 6 (46.12%), while the lowest was observed in region No. 1 (40.87%), representing a difference of more than 5 percentage points. This finding suggests that the spatial distribution of particles within the sample is not entirely uniform, and local particle aggregations may significantly affect particle density in specific regions. The obtained results indicate that, although the analyzed images were derived from the same sample, local variations in particle area and density were observed. This confirms that, when interpreting quantitative results, the heterogeneity of the examined materials must be taken into account, even when they originate from a single source. The same measurements were performed for the sample after recycling. The results are presented in Table 2.

The analysis of the obtained results indicates a relatively high consistency of the morphometric parameters of the particles. The mean particle count was 2682, with extreme values ranging from 2275 (No. 10) to 3018 (No. 2). The highest particle density was recorded in region No. 2, where the number of objects reached 3018, and the corresponding total area amounted to 591,514 px (5915.14 µ$m^{2}$), which also translated into the highest particle density in the entire dataset (9.51%). Conversely, the lowest particle count was observed in region No. 10, where 2275 particles with a total area of 449,639 px (4496.39 µ$m^{2}$) were identified. The particle density in this case was also the lowest, at 7.23%. The total particle area in individual images was proportional to their count. Most of the analyzed images exhibited comparable particle density, ranging from 7.23% to 9.51%, with a mean value of 8.30%. Such small differences indicate a high degree of sample homogeneity, while the observed deviations may be attributed to factors related to sample preparation for image analysis (including placement of a sample on a glass with a spatula from the vial). Therefore, the obtained results demonstrate that the examined sample exhibits stable morphometry within the analyzed image fields, with dominant values of particle count, total area, and density pointing to its high homogeneity (with the exception of region No. 10).

### 3.2. Analysis of Particle Area

**Figure 6:**
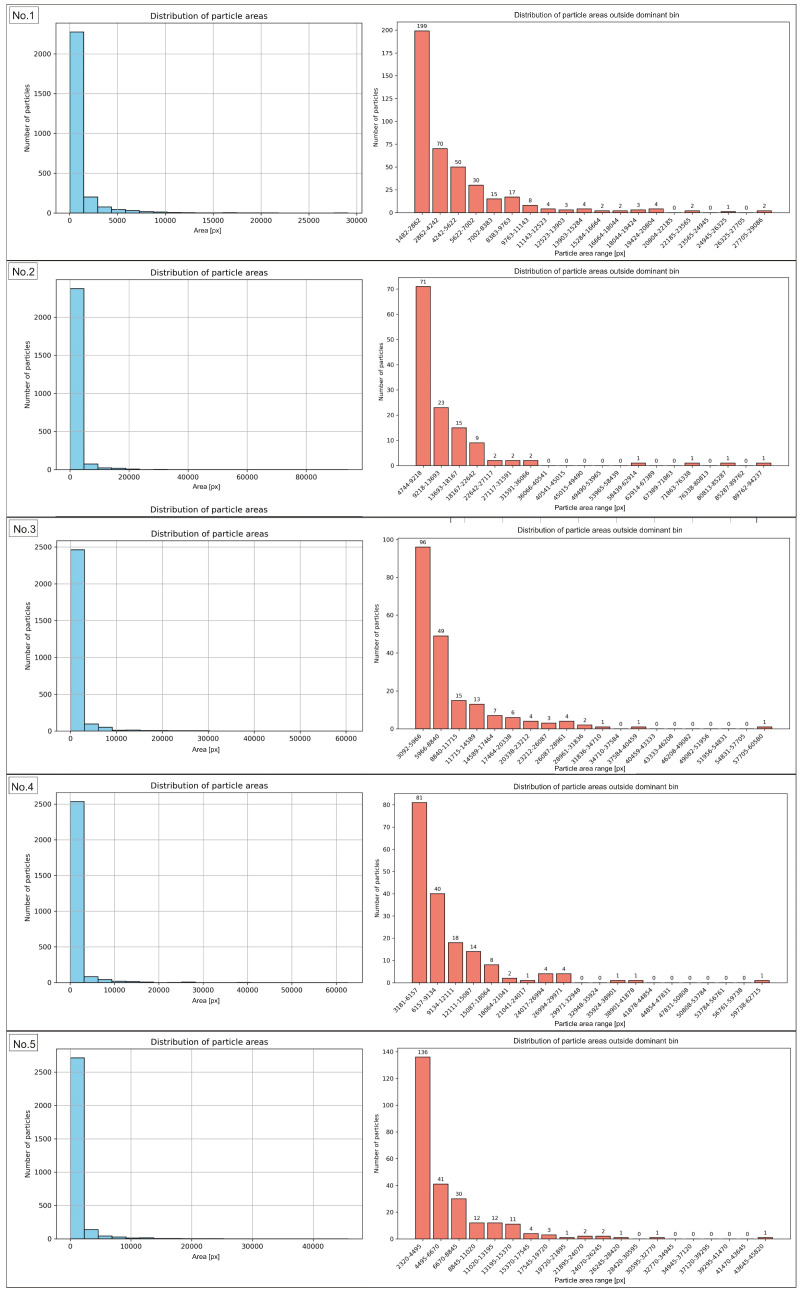
Histograms of the particle area distribution in the sample images before recycling (**left graphs**), along with distribution of particle areas outside dominant bin (**right graphs**) for images No. 1 to No. 5.

**Figure 7:**
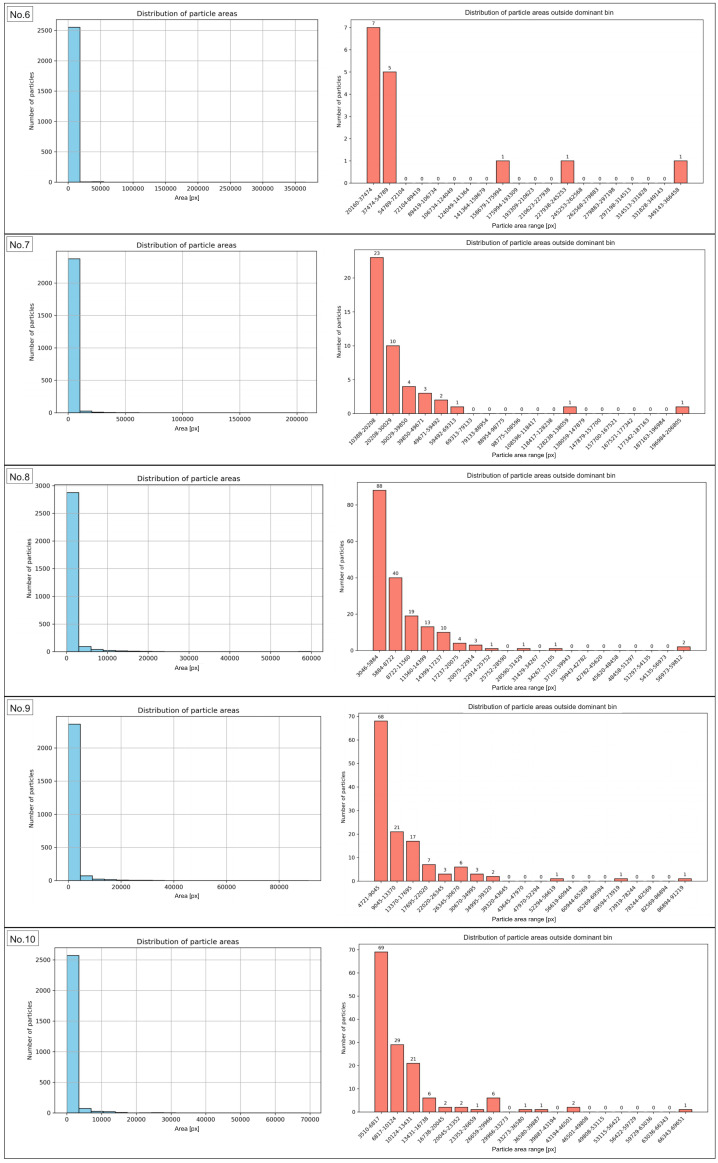
Histograms of the particle area distribution in the sample images before recycling (**left graphs**), along with distribution of particle areas outside dominant bin (**right graphs**) for images No. 6 to No. 10.

**Figure A1:**
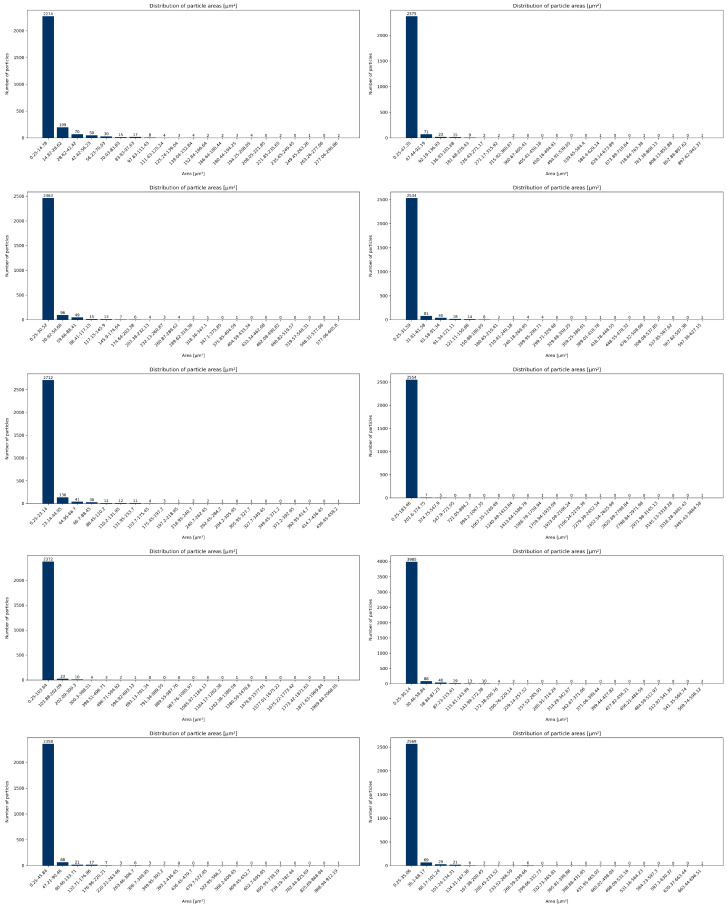
Histograms of the particle area distribution, expressed in µ$m^{2}$, in the sample images before recycling process.

In order to characterize the manufactured composite and determine which particle area fractions are dominant in the recycled sample, an analysis of particle areas in the sample images before and after the recycling process was conducted. The results are presented in the form of particle area distribution histograms. They show the frequency of particles occurring within specific area ranges, providing information about the structure of the studied material. This demonstrates which particle area classes dominate and indicates the degree of uniformity or variability in particle sizes. The peaks of the histogram represent the most frequently occurring areas, while a broad distribution reflects a heterogeneous mixture. The histograms of the particle area range for the sample before recycling are presented in Figure 6 and Figure 7 for regions No. 1 to No. 5, and No. 6 to No. 10, respectively. The same data, expressed in µ$m^{2}$, are presented in Figure A1.

Table 3 presents the range of particle area values most frequently observed in the analyzed images of the sample before recycling, the number of particles within this range, and their percentage share relative to all particles in the image.

**Figure 8:**
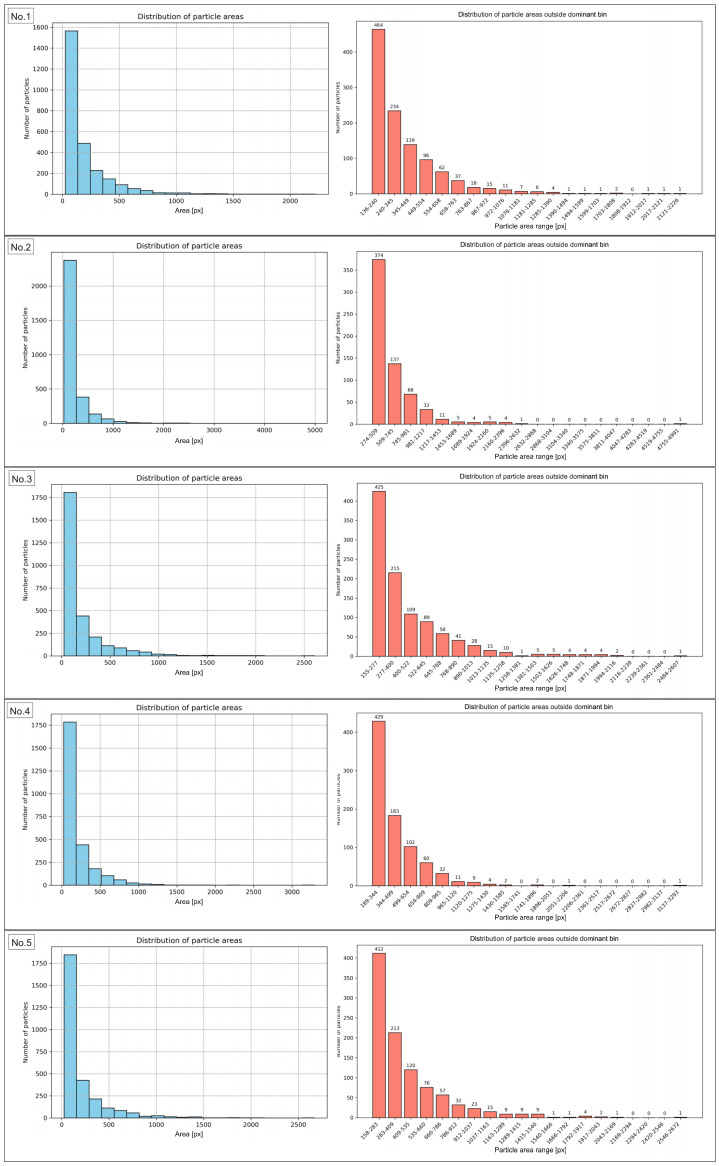
Histograms of the particle area distribution in the sample images after recycling process (**left graphs**) along with distribution of particle areas outside dominant bin (**right graphs**) for images No. 1 to No. 5.

**Figure 9:**
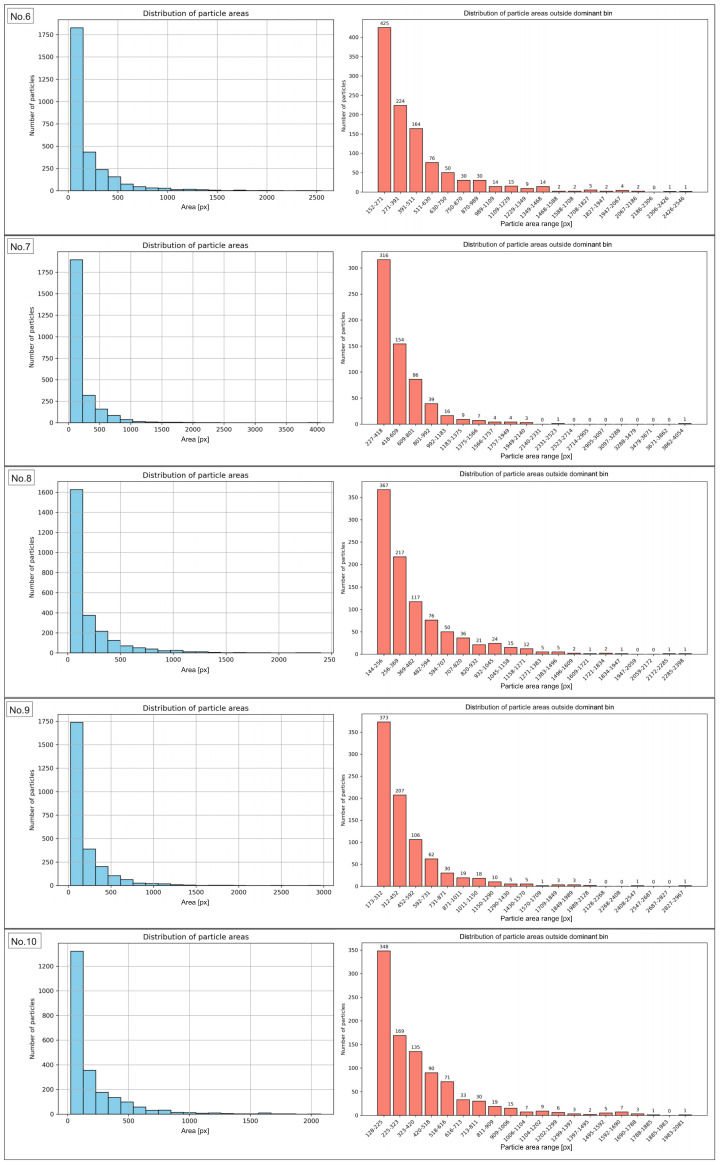
Histograms of the particle area distribution in the sample images after recycling process (**left graphs**), along with distribution of particle areas outside dominant bin (**right graphs**) for images No. 6 to No. 10.

**Figure A2:**
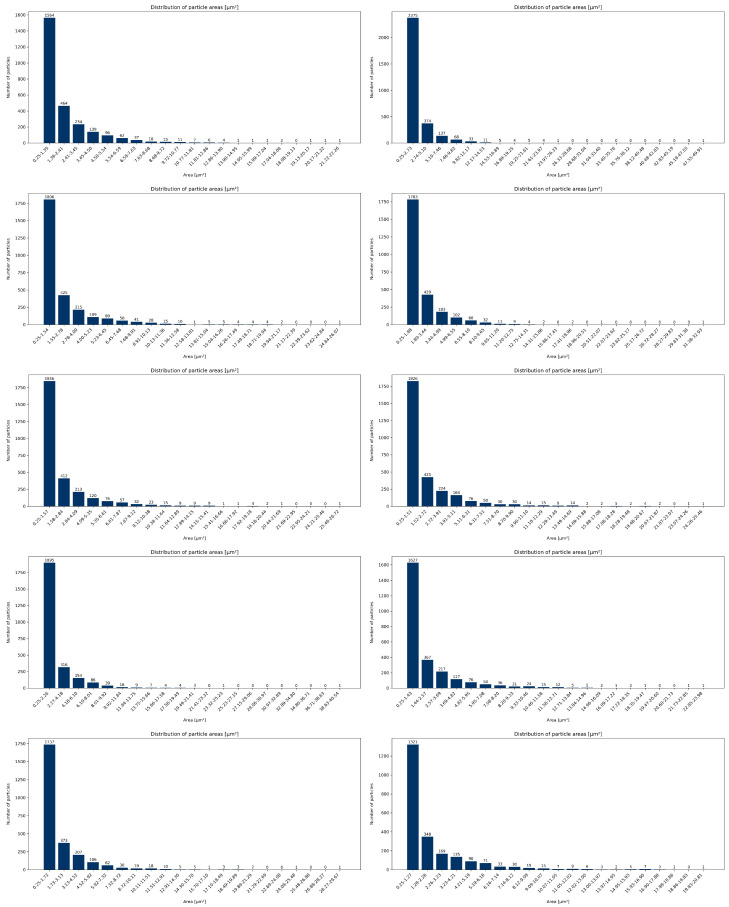
Histograms of the particle area distribution, expressed in µ$m^{2}$, in the sample images after recycling process.

The results of the morphometric analysis showed that the vast majority of particles in the examined sample fell within specific area ranges, confirming the high morphological uniformity of the material. The number of particles within the dominant area intervals ranged from 2274 to 2874, accounting for 84.54% to as much as 99.42% of the total particle population. This indicates that nearly all objects were characterized by areas within the established limits, while particles deviating from these ranges represented only a marginal share. The exact data for particles outside the range are presented in Tables S1–S10. Of particular importance was the finding that in area No. 6, the widest surface area range, from 25 to 18,346 px, was identified, encompassing 99.42% of the particles. The broadening of this interval resulted from the presence of a few larger objects. However, the majority of the population remained consolidated within this broad boundary. In contrast, region No. 1 showed the lowest proportion of particles falling within the most common surface range (84.54%), indicating greater local morphological variability compared to the other regions. The analysis of particle area distribution in regions 2 through 10, excluding region No. 1, demonstrated the stability and high reproducibility of the results. The share of particles in the dominant ranges exceeded 90%, and in most cases was approximately 95%. Such a distribution indicates the morphological consistency of the studied material. The obtained results clearly confirm that the particle sizes in the analyzed region are strongly concentrated within specific area ranges. The same analyses were conducted for the sample images after recycling. The results are presented in Figure 8, for images from No. 1 to No. 5, and in Figure 9, for images from No. 6 to No. 10, while Table 4 included numerical values. The same data, expressed in µ$m^{2}$, are presented in Figure A2.

Analysis of the presented results indicates that the distribution of the most frequently occurring particles in the examined sample is relatively homogeneous, with the share of particles within the dominant area ranges varying broadly from 58.07% to 78.69%. The highest value was recorded for region No. 2, in which 2375 particles were detected, accounting for 78.69% of their total number in the image. Such a high proportion indicates a clear dominance of particles with sizes corresponding to this range and a relatively small presence of particles with extreme dimensions. Conversely, the lowest share of particles within the dominant range was observed in regions No. 1 and No. 10, where it amounted to 58.69% and 58.07%, respectively. This means that in these images, a larger portion of the population was characterized by areas outside the analyzed range, suggesting a local increase in morphometric variability. In the remaining regions, the share of particles fell within the range from 63.05% (No. 6) to 74.75% (No. 7), indicating the stability and repeatability of the results in most analyzed cases. The numerical values for particles outside main range are presented in Tables S11–S20. It is worth emphasizing that all dominant area ranges started from the value of 25 px, while their upper limits varied between regions depending on the local particle distribution, ranging from 127 px (No. 10) to 273 px (No. 2). This indicates relatively small but noticeable differences in the maximum sizes of the most frequently occurring particles across individual image fields. Generally, the obtained results clearly confirm that the majority of particles in the examined sample fall within relatively narrow and repeatable area ranges, with the exceptions of regions No. 1 and No. 10.

## 4. Discussion

**Figure A3:**
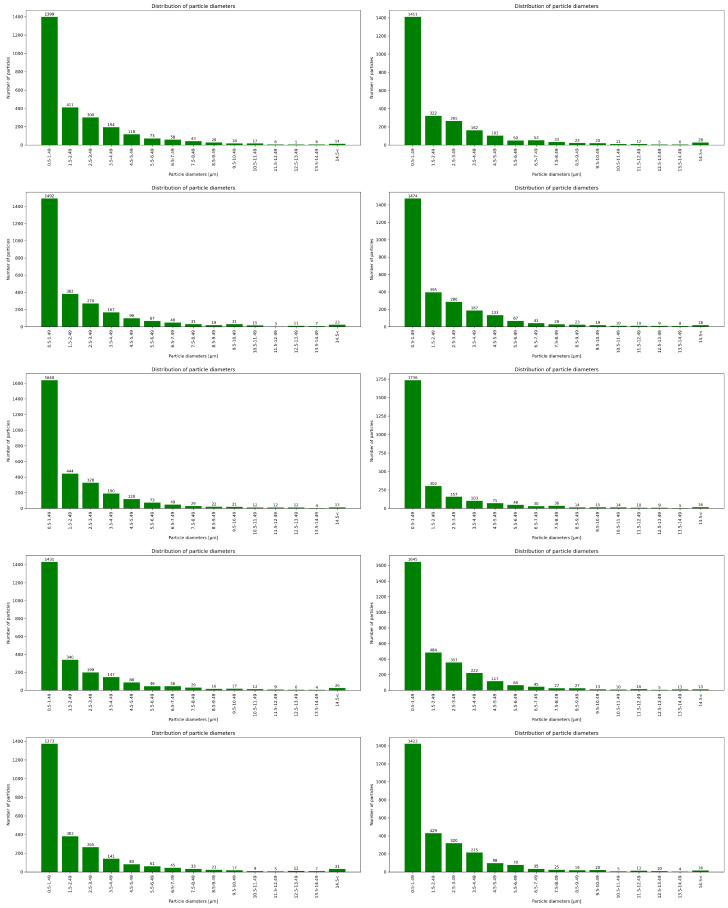
Histogram of the distribution of particle diameteres, expressed in µm, in the sample images before recycling process.

**Figure A4:**
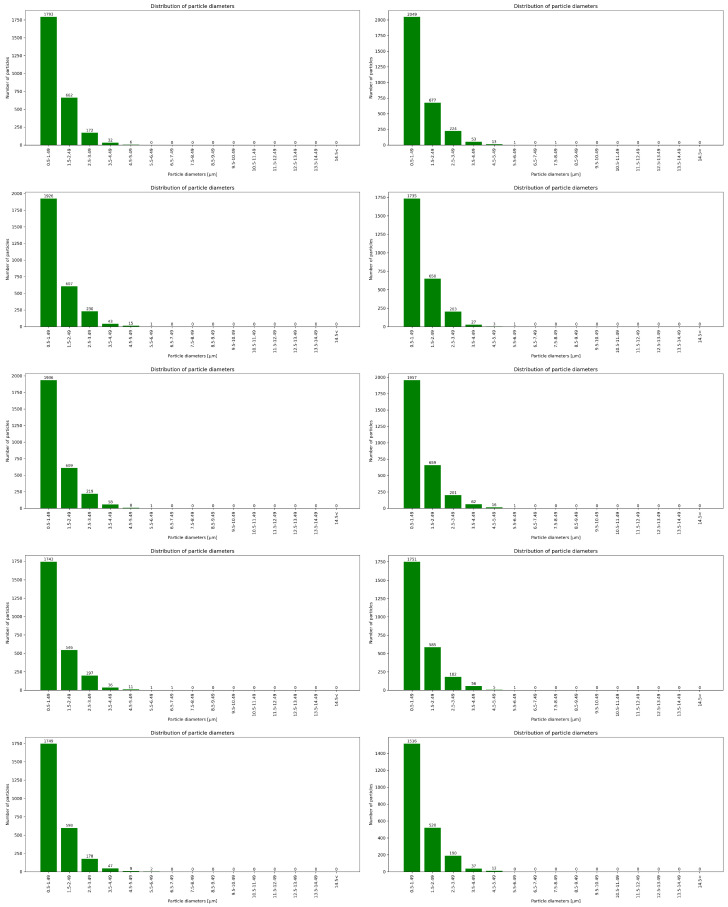
Histogram of the distribution of particle diameteres, expressed in µm, in the sample images after recycling process.

The comparison of data determined for the sample before and after the recycling process indicated that even though a number of particles remained at relatively the same level (2677 and 2682, accordingly for sample prior and after recycling process), the explicit decrease was stated for total area value and particle density. Consequently, the effectiveness of the recycling process could be confirmed. Before recycling, the average total particle area amounted to 2.69 million px, whereas after recycling it decreased to approximately 516 thousand px. This corresponds to nearly a fivefold reduction in surface coverage. Similarly, the average particle density significantly decreased from 43.30% to 8.30%. These results show that despite a comparable number of particles, the recycling process led to a substantial removal of bigger particles and agglomerates, resulting in a lowered area fraction of particles within the examined area, as particle size distribution histograms before recycling (Figure A3) and after recycling (Figure A4) confirm. Additionally, a pronounced narrowing of the density range was observed (from 40.87–46.12% before recycling to 7.23–9.51% after recycling). Post-recycling, variability between individual images was markedly reduced, indicating a more uniform distribution of smaller particles. The findings confirm that the applied recycling method was effective in recovering a significant number of particles. The examination of particle area distribution histograms before and after recycling enabled the evaluation of variations in particle areas and the determination of the degree of their concentration within specific size intervals. In samples analyzed prior to recycling, the vast majority of particles were located within dominant intervals. Their share ranged from 84.54% to 99.42%, with nine out of ten regions exceeding 90%. Post-recycling analysis revealed a marked decrease in the proportion of particles within the dominant area ranges, varying from 58.07% (regions No. 10) to 78.69% (regions No. 2).

The developed analysis revealed, that—taking into consideration mean values included in Table 1 and Table 2—the number of particles could be stated at a relatively similar level for both samples. Nevertheless, it is important to acknowledge that image analysis techniques, like any other technique, possess *inherent* limitations. It could encounter difficulties in accurately quantifying particle counts in the presence of agglomerates. Here, the image characterization exhibited some agglomerates (Figure 1), which, again, are a consequence of the composite’s functionality or, potentially, emphasizes the need for the further advancement of the manufacturing process. Despite this, the explicit difference is noticeable in the values of total area as well as particle density (as mentioned above), confirming the efficiency of the recycling protocol. Indeed, melting the paraffin matrix and adding turpentine facilitated phase separation (paraffin and tungsten particles); consequently, after centrifugation step, the tungsten microparticles gathered at the bottom of the vial. Finally, the residuals—tungsten microparticles that remained in the waste (i.e., paraffin, turpentine) above the sediment part—showed lower numbers of particle density (mean value 8.30%) in comparison to the manufactured composite sample (mean value of particle density 43.30%), which confirms the contribution of all subsequent steps in the recycling protocol.

## 5. Conclusions

This study refers to the quantitative analysis estimation of a recycling process utilizing commonly available and relatively straightforward tools—images acquired with a visible-light microscopy accompanied by computed processing (adaptive thresholding, smoothing step, etc.) Quantitative analysis of particle count, surface area, and density before and after the recycling process provided essential information on the morphology and homogeneity of the examined sample. Prior to recycling, the number of particles was relatively stable, while the total particle area and density exhibited greater variability, indicating local differences in particle size and agglomeration, confirming the heterogeneous structure of the material. The existence of agglomerates arises from the functionality of developed composites as the shape is influenced by hand-force; consequently, changes in shape lead to an alteration in the location of dispersed particles within matrix. The simplicity of the manufacturing procedure cannot be overlooked as it could play a role in agglomeration phenomena; thus, improvements (for instance, additional steps following cold mixing) may be worth exploring regarding their overall impact on the contribution of the presented analyses. After recycling, the particle count remained close to the initial state, whereas the total particle area and density decreased significantly the value of the total particles area reduced from 26,936.74 µ$m^{2}$ to 5161.78 µ$m^{2}$, while the primary particle density value of 43.30% decreased to 8.30% according to the samples before and after the recycling process. Based on the acquired microscopy images, it can be noticed that most particles exhibited a more uniform dispersion within wastes (melted paraffin and turpentine) and narrower size distribution, thus generally demonstrating the efficiency of the recycling process. However, minor deviations in the number of particles and their size were observed in some images. The results confirm that the recycling process allows for the recovery of larger particles and agglomerates. Moreover, quantitative analysis of particle area and density distribution provides a reliable method for assessing the effectiveness of the recycling process. These findings also highlight the importance of considering local heterogeneity when interpreting morphometric results, even for samples originating from a single source. Further research should be conducted focusing on the investigation of the quantitative estimation of recovered particles using alternative techniques (for instance, gravimetric methods), thus allowing for a holistic approach to the assessment of the efficiency of the recycling protocol.

## Figures and Tables

**Table 1:** Results of the image analysis showing the number of identified particles, their total area, and particle density for different regions of the sample before recycling.

Region Number	Number of Particles	Total Area [px]	Total Area [µm^2^]	Particle Density [%]
1	2690	2,542,596	25,425.96	40.87
2	2503	2,776,837	27,768.37	44.64
3	2665	2,711,547	27,115.47	43.59
4	2709	2,638,718	26,387.18	42.42
5	2969	2,560,910	25,609.10	41.17
6	2566	2,868,835	28,688.35	46.12
7	2417	2,847,912	28,479.12	45.78
8	3056	2,632,542	26,325.42	42.32
9	2488	2,808,102	28,081.02	45.14
10	2710	2,548,745	25,487.45	40.97
Mean	2677	2,693,674	26,936.74	43.30

**Table 2:** Results of the image analysis showing the number of identified particles, their total area, and particle density for different regions of the sample after recycling.

Region Number	Number of Particles	Total Area [px]	Total Area [µm^2^]	Particle Density [%]
1	2665	489,853	4898.53	7.87
2	3018	591,514	5915.14	9.51
3	2822	547,390	5473.90	8.80
4	2619	492,726	4927.26	7.92
5	2831	544,869	5448.69	8.76
6	2896	577,458	5774.58	9.28
7	2535	486,063	4860.63	7.81
8	2580	492,164	4921.64	7.91
9	2583	490,105	4901.05	7.88
10	2275	449,639	4496.39	7.23
Mean	2682	516,178	5161.78	8.30

**Table 3:** Particle area values for different regions of the sample before recycling (manufactured paraffin + 50 wt.% W).

Region Number	Particle Area Range of Predominant Particles [px]	Particle Area Range of Predominant Particles [µ$m^{2}$]	Particle Count Within Range	Percentage Share of Particles Within Range [%]
1	25–1478	0.25–14.78	2274	84.54
2	25–4735	0.25–47.35	2375	94.89
3	25–3052	0.25–30.52	2463	92.42
4	25–3159	0.25–31.59	2534	93.54
5	25–2314	0.25–23.14	2712	91.34
6	25–18,346	0.25–183.46	2551	99.42
7	25–10,364	0.25–103.64	2372	98.14
8	25–3014	0.25–30.14	2874	94.04
9	25–4584	0.25–45.84	2358	94.77
10	25–3506	0.25–35.06	2569	94.80

**Table 4:** Particle area values for different regions of the sample after recycling.

Region Number	Particle Area Range of Predominant Particles [px]	Particle Area Range of Predominant Particles [µ$m^{2}$]	Particle Count Within Range	Percentage Share of Particles Within Range [%]
1	25–135	0.25–1.35	1564	58.69
2	25–273	0.25–2.73	2375	78.69
3	25–154	0.25–1.54	1806	64.00
4	25–188	0.25–1.88	1783	68.08
5	25–157	0.25–1.57	1846	65.21
6	25–151	0.25–1.51	1826	63.05
7	25–226	0.25–2.26	1895	74.75
8	25–143	0.25–1.43	1627	63.06
9	25–172	0.25–1.72	1737	67.25
10	25–127	0.25–1.27	1321	58.07

## Data Availability

The original contributions presented in this study are included in the article/Supplementary Materials. Further inquiries can be directed to the corresponding authors.

## Supplementary Materials

The following supporting information can be downloaded at: https://www.mdpi.com/article/10.3390/ma18194519/s1, Table S1: Particle size distribution in the sample images before recycling—region 1; Table S2: Particle size distribution in the sample images before recycling—region 2; Table S3: Particle size distribution in the sample images before recycling—region 3; Table S4: Particle size distribution in the sample images before recycling—region 4; Table S5: Particle size distribution in the sample images before recycling—region 5; Table S6: Particle size distribution in the sample images before recycling—region 6; Table S7: Particle size distribution in the sample images before recycling—region 7; Table S8: Particle size distribution in the sample images before recycling—region 8; Table S9: Particle size distribution in the sample images before recycling—region 9; Table S10: Particle size distribution in the sample images before recycling—region 10; Table S11: Particle size distribution in the sample images after recycling—region 1; Table S12: Particle size distribution in the sample images after recycling—region 2; Table S13: Particle size distribution in the sample images after recycling—region 3; Table S14: Particle size distribution in the sample images after recycling—region 4; Table S15: Particle size distribution in the sample images after recycling—region 5; Table S16: Particle size distribution in the sample images after recycling—region 6; Table S17: Particle size distribution in the sample images after recycling—region 7; Table S18: Particle size distribution in the sample images after recycling—region 8; Table S19: Particle size distribution in the sample images after recycling—region 9; Table S20: Particle size distribution in the sample images after recycling—region 10.
