# Supplementary material for: Quantitative Evaluation of Composite Recyclability Using Visible-Light Microscopy and Image Processing Techniques
Source: Materials (Basel). 2025 Sep 28;18(19):4519. doi: 10.3390/ma18194519 (PMC12526482; doi:10.3390/ma18194519)
Supplement: Supplementary file 1 [file materials-18-04519-s001.zip › materials-3874550-supplementary.pdf]

Supplementary Materials: Quantitative evaluation of composite recyclability using visible-light microscopy and image processing techniques

Róża Dzierżak [1,†], Jolanta Sobczak [2,†], Gaweł Żyła [3,*] and Jacek Fal [3,*]

Table S1: Particle size distribution in the sample images before recycling - region 1.

Size range of particles [px]	Size range of particles [μm^2]	Particle count within range	Percentage share of particles within range [%]
25 – 1478	0.25 – 14.8	2274	84.54
1482 – 2862	14.8 – 28.6	199	7.40
2862 – 4242	28.6 – 42.4	70	2.60
4242 – 5623	42.4 – 56.2	50	1.86
5623 – 7003	56.2 – 70.0	30	1.12
7003 – 8383	70.0 – 83.8	15	0.56
8383 – 9763	83.8 – 97.6	17	0.63
9763 – 11143	97.6 – 111.4	8	0.30
11143 – 12524	111.4 – 125.2	4	0.15
12524 – 13904	125.2 – 139.0	3	0.11
13904 – 15284	139.0 – 152.8	4	0.15
15284 – 16664	152.8 – 166.6	2	0.07
16664 – 18044	166.6 – 180.4	2	0.07
18044 – 19425	180.4 – 194.2	3	0.11
19425 – 20805	194.2 – 208.0	4	0.15
20805 – 22185	208.0 – 221.9	0	0.00
22185 – 23565	221.9 – 235.7	2	0.07
23565 – 24945	235.7 – 249.5	0	0.00
24945 – 26326	249.5 – 263.3	1	0.04
26326 – 27706	263.3 – 277.1	0	0.00
27706 – 29086	277.1 – 290.9	2	0.07

Table S2: Particle size distribution in the sample images before recycling - region 2.

Area range of particles [px]	Area range of particles [μm^2]	Particle count within range	Percentage share of particles within range [%]
25 – 4735	0.25 – 47.4	2375	94.89
4744 – 9219	47.4 – 92.2	71	2.64
9219 – 13693	92.2 – 136.9	23	0.86
13693 – 18168	136.9 – 181.7	15	0.56
18168 – 22643	181.7 – 226.4	9	0.33
22643 – 27117	226.4 – 271.2	2	0.07
27117 – 31592	271.2 – 315.9	2	0.07
31592 – 36067	315.9 – 360.7	2	0.07
36067 – 40541	360.7 – 405.4	0	0.00
40541 – 45016	405.4 – 450.2	0	0.00
45016 – 49491	450.2 – 494.9	0	0.00
49491 – 53965	494.9 – 539.7	0	0.00
53965 – 58440	539.7 – 584.4	0	0.00
58440 – 62914	584.4 – 629.1	1	0.04
62914 – 67389	629.1 – 673.9	0	0.00
67389 – 71864	673.9 – 718.6	0	0.00
71864 – 76338	718.6 – 763.4	1	0.04
76338 – 80813	763.4 – 808.1	0	0.00
80813 – 85288	808.1 – 852.9	1	0.04
85288 – 89762	852.9 – 897.6	0	0.00
89762 – 94237	897.6 – 942.4	1	0.04

Table S3: Particle size distribution in the sample images before recycling - region 3.

Area range of particles [px]	Area range of particles [μm^2]	Particle count within range	Percentage share of particles within range [%]
25 – 3052	0.25 – 30.5	2463	92.42
3092 – 5966	30.9 – 59.7	96	3.57
5966 – 8841	59.7 – 88.4	49	1.82
8841 – 11715	88.4 – 117.2	15	0.56
11715 – 14590	117.2 – 145.9	13	0.48
14590 – 17464	145.9 – 174.6	7	0.26
17464 – 20338	174.6 – 203.4	6	0.22
20338 – 23213	203.4 – 232.1	4	0.15
23213 – 26087	232.1 – 260.9	3	0.11
26087 – 28962	260.9 – 289.6	4	0.15
28962 – 31836	289.6 – 318.4	2	0.07
31836 – 34710	318.4 – 347.1	1	0.04
34710 – 37585	347.1 – 375.8	0	0.00
37585 – 40459	375.8 – 404.6	1	0.04
40459 – 43334	404.6 – 433.3	0	0.00
43334 – 46208	433.3 – 462.1	0	0.00
46208 – 49082	462.1 – 490.8	0	0.00
49082 – 51957	490.8 – 519.6	0	0.00
51957 – 54831	519.6 – 548.3	0	0.00
54831 – 57706	548.3 – 577.1	0	0.00
57706 – 60580	577.1 – 605.8	1	0.04

Table S4: Particle size distribution in the sample images before recycling - region 4.

Area range of particles [px]	Area range of particles [μm^2]	Particle count within range	Percentage share of particles within range [%]
25 – 3159	0.25 – 31.6	2534	93.54
3181 – 6158	31.8 – 61.6	81	3.01
6158 – 9134	61.6 – 91.3	40	1.49
9134 – 12111	91.3 – 121.1	18	0.67
12111 – 15088	121.1 – 150.9	14	0.52
15088 – 18065	150.9 – 180.6	8	0.30
18065 – 21041	180.6 – 210.4	2	0.07
21041 – 24018	210.4 – 240.2	1	0.04
24018 – 26995	240.2 – 269.9	4	0.15
26995 – 29971	269.9 – 299.7	4	0.15
29971 – 32948	299.7 – 329.5	0	0.00
32948 – 35925	329.5 – 359.2	0	0.00
35925 – 38901	359.2 – 389.0	1	0.04
38901 – 41878	389.0 – 418.8	1	0.04
41878 – 44855	418.8 – 448.5	0	0.00
44855 – 47832	448.5 – 478.3	0	0.00
47832 – 50808	478.3 – 508.1	0	0.00
50808 – 53785	508.1 – 537.8	0	0.00
53785 – 56762	537.8 – 567.6	0	0.00
56762 – 59738	567.6 – 597.4	0	0.00
59738 – 62715	597.4 – 627.2	1	0.04

Table S5: Particle size distribution in the sample images before recycling - region 5.

Area range of particles [px]	Area range of particles [μm^2]	Particle count within range	Percentage share of particles within range [%]
25 – 2314	0.25 – 23.1	2712	91.34
2320 – 4495	23.2 – 45.0	136	5.06
4495 – 6670	45.0 – 66.7	41	1.52
6670 – 8845	66.7 – 88.5	30	1.12
8845 – 11020	88.5 – 110.2	12	0.45
11020 – 13195	110.2 – 132.0	12	0.45
13195 – 15370	132.0 – 153.7	11	0.41
15370 – 17545	153.7 – 175.5	4	0.15
17545 – 19720	175.5 – 197.2	3	0.11
19720 – 21895	197.2 – 219.0	1	0.04
21895 – 24070	219.0 – 240.7	2	0.07
24070 – 26245	240.7 – 262.5	2	0.07
26245 – 28420	262.5 – 284.2	1	0.04
28420 – 30595	284.2 – 306.0	0	0.00
30595 – 32770	306.0 – 327.7	1	0.04
32770 – 34945	327.7 – 349.5	0	0.00
34945 – 37120	349.5 – 371.2	0	0.00
37120 – 39295	371.2 – 393.0	0	0.00
39295 – 41470	393.0 – 414.7	0	0.00
41470 – 43645	414.7 – 436.5	0	0.00
43645 – 45820	436.5 – 458.2	1	0.04

Table S6: Particle size distribution in the sample images before recycling - region 6.

Area range of particles [px]	Area range of particles [μm^2]	Particle count within range	Percentage share of particles within range [%]
25 – 18346	0.25 – 183.5	2554	99.42
20160 – 37475	201.6 – 374.7	7	0.26
37475 – 54790	374.7 – 547.9	5	0.19
54790 – 72105	547.9 – 721.0	0	0.00
72105 – 89420	721.0 – 894.2	0	0.00
89420 – 106735	894.2 – 1067.3	0	0.00
106735 – 124049	1067.3 – 1240.5	0	0.00
124049 – 141364	1240.5 – 1413.6	0	0.00
141364 – 158679	1413.6 – 1586.8	0	0.00
158679 – 175994	1586.8 – 1759.9	1	0.04
175994 – 193309	1759.9 – 1933.1	0	0.00
193309 – 210624	1933.1 – 2106.2	0	0.00
210624 – 227939	2106.2 – 2279.4	0	0.00
227939 – 245254	2279.4 – 2452.5	1	0.04
245254 – 262569	2452.5 – 2625.7	0	0.00
262569 – 279884	2625.7 – 2798.8	0	0.00
279884 – 297198	2798.8 – 2972.0	0	0.00
297198 – 314513	2972.0 – 3145.1	0	0.00
314513 – 331828	3145.1 – 3318.3	0	0.00
331828 – 349143	3318.3 – 3491.4	0	0.00
349143 – 366458	3491.4 – 3664.6	1	0.04

Table S7: Particle size distribution in the sample images before recycling - region 7.

Area range of particles [px]	Area range of particles [μm^2]	Particle count within range	Percentage share of particles within range [%]
25 – 10364	0.25 – 103.6	2372	98.14
10388 – 20209	103.9 – 202.1	23	0.86
20209 – 30030	202.1 – 300.3	10	0.37
30030 – 39851	300.3 – 398.5	4	0.15
39851 – 49671	398.5 – 496.7	3	0.11
49671 – 59492	496.7 – 594.9	2	0.07
59492 – 69313	594.9 – 693.1	1	0.04
69313 – 79134	693.1 – 791.3	0	0.00
79134 – 88955	791.3 – 889.5	0	0.00
88955 – 98776	889.5 – 987.8	0	0.00
98776 – 108597	987.8 – 1086.0	0	0.00
108597 – 118417	1086.0 – 1184.2	0	0.00
118417 – 128238	1184.2 – 1282.4	0	0.00
128238 – 138059	1282.4 – 1380.6	1	0.04
138059 – 147880	1380.6 – 1478.8	0	0.00
147880 – 157701	1478.8 – 1577.0	0	0.00
157701 – 167522	1577.0 – 1675.2	0	0.00
167522 – 177342	1675.2 – 1773.4	0	0.00
177342 – 187163	1773.4 – 1871.6	0	0.00
187163 – 196984	1871.6 – 1969.8	0	0.00
196984 – 206805	1969.8 – 2068.1	1	0.04

Table S8: Particle size distribution in the sample images before recycling - region 8.

Area range of particles [px]	Area range of particles [μm^2]	Particle count within range	Percentage share of particles within range [%]
25 – 3014	0.25 – 30.1	3985	94.04
3046 – 5884	30.5 – 58.8	88	3.27
5884 – 8723	58.8 – 87.2	40	1.49
8723 – 11561	87.2 – 115.6	19	0.71
11561 – 14399	115.6 – 144.0	13	0.48
14399 – 17238	144.0 – 172.4	10	0.37
17238 – 20076	172.4 – 200.8	4	0.15
20076 – 22914	200.8 – 229.1	3	0.11
22914 – 25752	229.1 – 257.5	1	0.04
25752 – 28591	257.5 – 285.9	0	0.00
28591 – 31429	285.9 – 314.3	1	0.04
31429 – 34267	314.3 – 342.7	0	0.00
34267 – 37106	342.7 – 371.1	1	0.04
37106 – 39944	371.1 – 399.4	0	0.00
39944 – 42782	399.4 – 427.8	0	0.00
42782 – 45621	427.8 – 456.2	0	0.00
45621 – 48459	456.2 – 484.6	0	0.00
48459 – 51297	484.6 – 513.0	0	0.00
51297 – 54135	513.0 – 541.4	0	0.00
54135 – 56974	541.4 – 569.7	0	0.00
56974 – 59812	569.7 – 598.1	2	0.07

Table S9: Particle size distribution in the sample images before recycling - region 9.

Area range of particles [px]	Area range of particles [μm^2]	Particle count within range	Percentage share of particles within range [%]
25 – 4584	0.25 – 45.8	2358	94.77
4721 – 9046	47.2 – 90.5	68	2.53
9046 – 13371	90.5 – 133.7	21	0.78
13371 – 17696	133.7 – 177.0	17	0.63
17696 – 22021	177.0 – 220.2	7	0.26
22021 – 26346	220.2 – 263.5	3	0.11
26346 – 30670	263.5 – 306.7	6	0.22
30670 – 34995	306.7 – 350.0	3	0.11
34995 – 39320	350.0 – 393.2	2	0.07
39320 – 43645	393.2 – 436.5	0	0.00
43645 – 47970	436.5 – 479.7	0	0.00
47970 – 52295	479.7 – 522.9	0	0.00
52295 – 56620	522.9 – 566.2	1	0.04
56620 – 60945	566.2 – 609.4	0	0.00
60945 – 65270	609.4 – 652.7	0	0.00
65270 – 69595	652.7 – 695.9	0	0.00
69595 – 73919	695.9 – 739.2	1	0.04
73919 – 78244	739.2 – 782.4	0	0.00
78244 – 82569	782.4 – 825.7	0	0.00
82569 – 86894	825.7 – 868.9	0	0.00
86894 – 91219	868.9 – 912.2	1	0.04

Table S10: Particle size distribution in the sample images before recycling - region 10.

**Area range of particles [px]**	**Area range of particles [μm^2]**	**Particle count within range**	**Percentage share of particles within range [%]**
25 – 3506	0.25 – 35.1	2569	94.8
3510 – 6817	35.1 – 68.2	69	2.57
6817 – 10124	68.2 – 101.2	29	1.08
10124 – 13431	101.2 – 134.3	21	0.78
13431 – 16738	134.3 – 167.4	6	0.22
16738 – 20045	167.4 – 200.5	2	0.07
20045 – 23352	200.5 – 233.5	2	0.07
23352 – 26659	233.5 – 266.6	1	0.04
26659 – 29966	266.6 – 299.7	6	0.22
29966 – 33273	299.7 – 332.7	0	0.00
33273 – 36581	332.7 – 365.8	1	0.04
36581 – 39888	365.8 – 398.9	1	0.04
39888 – 43195	398.9 – 431.9	0	0.00
43195 – 46502	431.9 – 465.0	2	0.07
46502 – 49809	465.0 – 498.1	0	0.00
49809 – 53116	498.1 – 531.2	0	0.00
53116 – 56423	531.2 – 564.2	0	0.00
56423 – 59730	564.2 – 597.3	0	0.00
59730 – 63037	597.3 – 630.4	0	0.00
63037 – 66344	630.4 – 663.4	0	0.00
66344 – 69651	663.4 – 696.5	1	0.04

Table S11: Particle size distribution in the sample images after recycling - region 1.

textbfArea range of particles [px]	**Area range of particles [μm^2]**	**Particle count within range**	**Percentage share of particles within range [%]**
25 – 135	0.25 – 1.4	1564	58.69
136 – 240.5	1.4 – 2.4	464	17.41
240.5 – 345	2.4 – 3.5	234	8.78
345 – 449.5	3.5 – 4.5	139	5.22
449.5 – 554	4.5 – 5.5	96	3.60
554 – 658.5	5.5 – 6.6	62	2.33
658.5 – 763	6.6 – 7.6	37	1.39
763 – 867.5	7.6 – 8.7	18	0.68
867.5 – 972	8.7 – 9.7	15	0.56
972 – 1076.5	9.7 – 10.8	11	0.41
1076.5 – 1181	10.8 – 11.8	7	0.26
1181 – 1285.5	11.8 – 12.9	6	0.23
1285.5 – 1390	12.9 – 13.9	4	0.15
1390 – 1494.5	13.9 – 14.9	1	0.04
1494.5 – 1599	14.9 – 16.0	1	0.04
1599 – 1703.5	16.0 – 17.0	1	0.04
1703.5 – 1808	17.0 – 18.1	2	0.08
1808 – 1912.5	18.1 – 19.1	0	0.00
1912.5 – 2017	19.1 – 20.2	1	0.04
2017 – 2121.5	20.2 – 21.2	1	0.04
2121.5 – 2226	21.2 – 22.3	1	0.04

Table S12: Particle size distribution in the sample images after recycling - region 2.

Area range of particles [px]	Area range of particles [μm^2]	Particle count within range	Percentage share of particles within range [%]
25 – 273	0.25 – 2.7	2375	78.69
274 – 509.85	2.7 – 5.1	374	12.39
509.85 – 745.7	5.1 – 7.5	137	4.54
745.7 – 981.55	7.5 – 9.8	68	2.25
981.55 – 1217.4	9.8 – 12.2	33	1.09
1217.4 – 1453.25	12.2 – 14.5	11	0.36
1453.25 – 1689.1	14.5 – 16.9	5	0.17
1689.1 – 1924.95	16.9 – 19.2	4	0.13
1924.95 – 2160.8	19.2 – 21.6	5	0.17
2160.8 – 2396.65	21.6 – 24.0	4	0.13
2396.65 – 2632.5	24.0 – 26.3	1	0.03
2632.5 – 2868.35	26.3 – 28.7	0	0.00
2868.35 – 3104.2	28.7 – 31.0	0	0.00
3104.2 – 3340.05	31.0 – 33.4	0	0.00
3340.05 – 3575.9	33.4 – 35.8	0	0.00
3575.9 – 3811.75	35.8 – 38.1	0	0.00
3811.75 – 4047.6	38.1 – 40.5	0	0.00
4047.6 – 4283.45	40.5 – 42.8	0	0.00
4283.45 – 4519.3	42.8 – 45.2	0	0.00
4519.3 – 4755.15	45.2 – 47.6	0	0.00
4755.15 – 4991	47.6 – 49.9	1	0.03

Table S13: Particle size distribution in the sample images after recycling - region 3.

Area range of particles [px]	Area range of particles [μm^2]	Particle count within range	Percentage share of particles within range [%]
25 – 154	0.25 – 1.5	1806	64.00
155 – 277.6	1.6 – 2.8	425	15.06
277.6 – 400.2	2.8 – 4.0	215	7.62
400.2 – 522.8	4.0 – 5.2	109	3.86
522.8 – 645.4	5.2 – 6.5	89	3.15
645.4 – 768	6.5 – 7.7	58	2.06
768 – 890.6	7.7 – 8.9	41	1.45
890.6 – 1013.2	8.9 – 10.1	28	0.99
1013.2 – 1135.8	10.1 – 11.4	15	0.53
1135.8 – 1258.4	11.4 – 12.6	10	0.35
1258.4 – 1381	12.6 – 13.8	1	0.04
1381 – 1503.6	13.8 – 15.0	5	0.18
1503.6 – 1626.2	15.0 – 16.3	5	0.18
1626.2 – 1748.8	16.3 – 17.5	4	0.14
1748.8 – 1871.4	17.5 – 18.7	4	0.14
1871.4 – 1994	18.7 – 19.9	4	0.14
1994 – 2116.6	19.9 – 21.2	2	0.07
2116.6 – 2239.2	21.2 – 22.4	0	0.00
2239.2 – 2361.8	22.4 – 23.6	0	0.00
2361.8 – 2484.4	23.6 – 24.8	0	0.00
2484.4 – 2607	24.8 – 26.1	1	0.04

Table S14: Particle size distribution in the sample images after recycling - region 4.

Area range of particles [px]	Area range of particles [μm^2]	Particle count within range	Percentage share of particles within range [%]
25 – 188	0.25 – 1.9	1783	68.08
189 – 344.2	1.9 – 3.4	429	16.38
344.2 – 499.4	3.4 – 5.0	183	6.99
499.4 – 654.6	5.0 – 6.5	102	3.89
654.6 – 809.8	6.5 – 8.1	60	2.29
809.8 – 965	8.1 – 9.7	32	1.22
965 – 1120.2	9.7 – 11.2	11	0.42
1120.2 – 1275.4	11.2 – 12.8	9	0.34
1275.4 – 1430.6	12.8 – 14.3	4	0.15
1430.6 – 1585.8	14.3 – 15.9	2	0.08
1585.8 – 1741	15.9 – 17.4	0	0.00
1741 – 1896.2	17.4 – 19.0	2	0.08
1896.2 – 2051.4	19.0 – 20.5	0	0.00
2051.4 – 2206.6	20.5 – 22.1	1	0.04
2206.6 – 2361.8	22.1 – 23.6	0	0.00
2361.8 – 2517	23.6 – 25.2	0	0.00
2517 – 2672.2	25.2 – 26.7	0	0.00
2672.2 – 2827.4	26.7 – 28.3	0	0.00
2827.4 – 2982.6	28.3 – 29.8	0	0.00
2982.6 – 3137.8	29.8 – 31.4	0	0.00
3137.8 – 3293	31.4 – 32.9	1	0.04

Table S15: Particle size distribution in the sample images after recycling - region 5.

Area range of particles [px]	Area range of particles [μm^2]	Particle count within range	Percentage share of particles within range [%]
25 – 157	0.25 – 1.6	1846	65.21
158 – 283.7	1.6 – 2.8	412	14.55
283.7 – 409.4	2.8 – 4.1	213	7.52
409.4 – 535.1	4.1 – 5.4	120	4.24
535.1 – 660.8	5.4 – 6.6	76	2.68
660.8 – 786.5	6.6 – 7.9	57	2.01
786.5 – 912.2	7.9 – 9.1	32	1.13
912.2 – 1037.9	9.1 – 10.4	23	0.81
1037.9 – 1163.6	10.4 – 11.6	15	0.53
1163.6 – 1289.3	11.6 – 12.9	9	0.32
1289.3 – 1415	12.9 – 14.2	9	0.32
1415 – 1540.7	14.2 – 15.4	9	0.32
1540.7 – 1666.4	15.4 – 16.7	1	0.04
1666.4 – 1792.1	16.7 – 17.9	1	0.04
1792.1 – 1917.8	17.9 – 19.2	4	0.14
1917.8 – 2043.5	19.2 – 20.4	2	0.07
2043.5 – 2169.2	20.4 – 21.7	1	0.04
2169.2 – 2294.9	21.7 – 22.9	0	0.00
2294.9 – 2420.6	22.9 – 24.2	0	0.00
2420.6 – 2546.3	24.2 – 25.5	0	0.00
2546.3 – 2672	25.5 – 26.7	1	0.04

Table S16: Particle size distribution in the sample images after recycling - region 6.

Area range of particles [px]	Area range of particles [μm^2]	Particle count within range	Percentage share of particles within range [%]
25 – 151	0.25 – 1.5	1826	63.05
152 – 271.7	1.5 – 2.7	425	14.68
271.7 – 391.4	2.7 – 3.9	224	7.73
391.4 – 511.1	3.9 – 5.1	164	5.66
511.1 – 630.8	5.1 – 6.3	76	2.62
630.8 – 750.5	6.3 – 7.5	50	1.73
750.5 – 870.2	7.5 – 8.7	30	1.04
870.2 – 989.9	8.7 – 9.9	30	1.04
989.9 – 1109.6	9.9 – 11.1	14	0.48
1109.6 – 1229.3	11.1 – 12.3	15	0.52
1229.3 – 1349	12.3 – 13.5	9	0.31
1349 – 1468.7	13.5 – 14.7	14	0.48
1468.7 – 1588.4	14.7 – 15.9	2	0.07
1588.4 – 1708.1	15.9 – 17.1	2	0.07
1708.1 – 1827.8	17.1 – 18.3	5	0.17
1827.8 – 1947.5	18.3 – 19.5	2	0.07
1947.5 – 2067.2	19.5 – 20.7	4	0.14
2067.2 – 2186.9	20.7 – 21.9	2	0.07
2186.9 – 2306.6	21.9 – 23.1	0	0.00
2306.6 – 2426.3	23.1 – 24.3	1	0.03
2426.3 – 2546	24.3 – 25.5	1	0.03

Table S17: Particle size distribution in the sample images after recycling - region 7.

Area range of particles [px]	Area range of particles [μm^2]	Particle count within range	Percentage share of particles within range [%]
25 – 226	0.25 – 2.3	1895	74.75
227 – 418.35	2.3 – 4.2	316	12.47
418.35 – 609.7	4.2 – 6.1	154	6.07
609.7 – 801.05	6.1 – 8.0	86	3.39
801.05 – 992.4	8.0 – 9.9	39	1.54
992.4 – 1183.75	9.9 – 11.8	16	0.63
1183.75 – 1375.1	11.8 – 13.8	9	0.36
1375.1 – 1566.45	13.8 – 15.7	7	0.28
1566.45 – 1757.8	15.7 – 17.6	4	0.16
1757.8 – 1949.15	17.6 – 19.5	4	0.16
1949.15 – 2140.5	19.5 – 21.4	3	0.12
2140.5 – 2331.85	21.4 – 23.3	0	0.00
2331.85 – 2523.2	23.3 – 25.2	1	0.04
2523.2 – 2714.55	25.2 – 27.1	0	0.00
2714.55 – 2905.9	27.1 – 29.1	0	0.00
2905.9 – 3097.25	29.1 – 31.0	0	0.00
3097.25 – 3288.6	31.0 – 32.9	0	0.00
3288.6 – 3479.95	32.9 – 34.8	0	0.00
3479.95 – 3671.3	34.8 – 36.7	0	0.00
3671.3 – 3862.65	36.7 – 38.6	0	0.00
3862.65 – 4054	38.6 – 40.5	1	0.04

Table S18: Particle size distribution in the sample images after recycling - region 8.

Area range of particles [px]	Area range of particles [μm^2]	Particle count within range	Percentage share of particles within range [%]
25 – 143	0.25 – 1.4	1627	63.06
144 – 256.7	1.4 – 2.6	367	14.22
256.7 – 369.4	2.6 – 3.7	217	8.41
369.4 – 482.1	3.7 – 4.8	117	4.53
482.1 – 594.8	4.8 – 5.9	76	2.95
594.8 – 707.5	5.9 – 7.1	50	1.94
707.5 – 820.2	7.1 – 8.2	36	1.40
820.2 – 932.9	8.2 – 9.3	21	0.81
932.9 – 1045.6	9.3 – 10.5	24	0.93
1045.6 – 1158.3	10.5 – 11.6	15	0.58
1158.3 – 1271	11.6 – 12.7	12	0.47
1271 – 1383.7	12.7 – 13.8	5	0.19
1383.7 – 1496.4	13.8 – 15.0	5	0.19
1496.4 – 1609.1	15.0 – 16.1	2	0.08
1609.1 – 1721.8	16.1 – 17.2	1	0.04
1721.8 – 1834.5	17.2 – 18.3	2	0.08
1834.5 – 1947.2	18.3 – 19.5	1	0.04
1947.2 – 2059.9	19.5 – 20.6	0	0.00
2059.9 – 2172.6	20.6 – 21.7	0	0.00
2172.6 – 2285.3	21.7 – 22.9	1	0.04
2285.3 – 2398	22.9 – 24.0	1	0.04

Table S19: Particle size distribution in the sample images after recycling - region 9.

Area range of particles [px]	Area range of particles [μm^2]	Particle count within range	Percentage share of particles within range [%]
25 – 172	0.25 – 1.7	1737	67.25
173 – 312.7	1.7 – 3.1	373	14.44
312.7 – 452.4	3.1 – 4.5	207	8.01
452.4 – 592.1	4.5 – 5.9	106	4.10
592.1 – 731.8	5.9 – 7.3	62	2.40
731.8 – 871.5	7.3 – 8.7	30	1.16
871.5 – 1011.2	8.7 – 10.1	19	0.74
1011.2 – 1150.9	10.1 – 11.5	18	0.70
1150.9 – 1290.6	11.5 – 12.9	10	0.39
1290.6 – 1430.3	12.9 – 14.3	5	0.19
1430.3 – 1570	14.3 – 15.7	5	0.19
1570 – 1709.7	15.7 – 17.1	1	0.04
1709.7 – 1849.4	17.1 – 18.5	3	0.12
1849.4 – 1989.1	18.5 – 19.9	3	0.12
1989.1 – 2128.8	19.9 – 21.3	2	0.08
2128.8 – 2268.5	21.3 – 22.7	0	0.00
2268.5 – 2408.2	22.7 – 24.1	0	0.00
2408.2 – 2547.9	24.1 – 25.5	1	0.04
2547.9 – 2687.6	25.5 – 26.9	0	0.00
2687.6 – 2827.3	26.9 – 28.3	0	0.00
2827.3 – 2967	28.3 – 29.7	1	0.04

Table S20: Particle size distribution in the sample images after recycling - region 10.

Area range of particles [px]	Area range of particles [μm^2]	Particle count within range	Percentage share of particles within range [%]
25 – 127	0.25 – 1.3	1321	58.07
128 – 225.65	1.3 – 2.3	348	15.30
225.65 – 323.3	2.3 – 3.2	169	7.43
323.3 – 420.95	3.2 – 4.2	135	5.93
420.95 – 518.6	4.2 – 5.2	90	3.96
518.6 – 616.25	5.2 – 6.2	71	3.12
616.25 – 713.9	6.2 – 7.1	33	1.45
713.9 – 811.55	7.1 – 8.1	30	1.32
811.55 – 909.2	8.1 – 9.1	19	0.84
909.2 – 1006.85	9.1 – 10.1	15	0.66
1006.85 – 1104.5	10.1 – 11.0	7	0.31
1104.5 – 1202.15	11.0 – 12.0	9	0.40
1202.15 – 1299.8	12.0 – 13.0	6	0.26
1299.8 – 1397.45	13.0 – 14.0	3	0.13
1397.45 – 1495.1	14.0 – 15.0	2	0.09
1495.1 – 1592.75	15.0 – 15.9	5	0.22
1592.75 – 1690.4	15.9 – 16.9	7	0.31
1690.4 – 1788.05	16.9 – 17.9	3	0.13
1788.05 – 1885.7	17.9 – 18.9	1	0.04
1885.7 – 1983.35	18.9 – 19.8	0	0.00
1983.35 – 2081	19.8 – 20.8	1	0.04

Disclaimer/Publisher's Note: The statements, opinions and data contained in all publications are solely those of the individual author(s) and contributor(s) and not of MDPI and/or the editor(s). MDPI and/or the editor(s) disclaim responsibility for any injury to people or property resulting from any ideas, methods, instructions or products referred to in the content.
